# Multi-Temporal and Spectral Analysis of High-Resolution Hyperspectral Airborne Imagery for Precision Agriculture: Assessment of Wheat Grain Yield and Grain Protein Content

**DOI:** 10.3390/rs10060930

**Published:** 2018-06-12

**Authors:** Francelino A. Rodrigues, Gerald Blasch, Pierre Defourny, J. Ivan Ortiz-Monasterio, Urs Schulthess, Pablo J. Zarco-Tejada, James A. Taylor, Bruno Gérard

**Affiliations:** 1 International Maize and Wheat Improvement Center—CIMMYT, Texcoco 56237, Mexico; I.Ortiz-Monasterio@cgiar.org (J.I.O.-M.); B.Gerard@cgiar.org (B.G.); 2 Food and Rural Development, School of Agriculture, Newcastle University, Newcastle NE1 7RU, UK; Gerald.Blasch@newcastle.ac.uk (G.B.); James.Taylor6@newcastle.ac.uk (J.A.T.); 3 Earth and Life Institute, Université Catholique de Louvain, Croix du Sud L5.07.16, B-1348 Louvain-la-Neuve, Belgium; Pierre.Defourny@uclouvain.be; 4 International Maize and Wheat Improvement Center—CIMMYT, Henan Agricultural University, 63 Nongye Road, Zhengzhou 450002, Henan, China; U.Schulthess@cgiar.org; 5 Instituto de Agricultura Sostenible (IAS), Consejo Superior de Investigaciones Científicas (CSIC), 14004 Cordoba, Spain; Pablo.Zarco@csic.es

**Keywords:** narrow-band indices, normalized difference spectral index, spatial-temporal variability, within-field variability, principal component analysis, time series

## Abstract

This study evaluates the potential of high resolution hyperspectral airborne imagery to capture within-field variability of durum wheat grain yield (GY) and grain protein content (GPC) in two commercial fields in the Yaqui Valley (northwestern Mexico). Through a weekly/biweekly airborne flight campaign, we acquired 10 mosaics with a micro-hyperspectral Vis-NIR imaging sensor ranging from 400–850 nanometres (nm). Just before harvest, 114 georeferenced grain samples were obtained manually. Using spectral exploratory analysis, we calculated narrow-band physiological spectral indices—normalized difference spectral index (NDSI) and ratio spectral index (RSI)—from every single hyperspectral mosaic using complete two by two combinations of wavelengths. We applied two methods for the multi-temporal hyperspectral exploratory analysis: (a) Temporal Principal Component Analysis (tPCA) on wavelengths across all images and (b) the integration of vegetation indices over time based on area under the curve (AUC) calculations. For GY, the best R^2^ (0.32) were found using both the spectral (NDSI—*Ri*, 750 to 840 nm and *Rj, ±*720–736 nm) and the multi-temporal AUC exploratory analysis (EVI and OSAVI through AUC) methods. For GPC, all exploratory analysis methods tested revealed (a) a low to very low coefficient of determination (R^2^ *≤* 0.21), (b) a relatively low overall prediction error (RMSE: 0.45–0.49%), compared to results from other literature studies, and (c) that the spectral exploratory analysis approach is slightly better than the multi-temporal approaches, with early season NDSI of 700 with 574 nm and late season NDSI of 707 with 523 nm as the best indicators. Using residual maps from the regression analyses of NDSIs and GPC, we visualized GPC within-field variability and showed that up to 75% of the field area could be mapped with relatively good predictability (residual class: *−*0.25 to 0.25%), therefore showing the potential of remote sensing imagery to capture the within-field variation of GPC under conventional agricultural practices.

## 1 Introduction

Wheat (*Triticum* sp.) is one of the three most important cereals produced worldwide, along with maize (*Zea mays*) and rice (*Oryza* sp.). It is also one of the most important crops in Mexico, grown on more than 723,559 ha in 2016, with average yields of 5.3 Mg ha*^−1^* [1]. Crop management is key to profitable and sustainable wheat production and must be able to respond to spatial and temporal variability in soil and climate with the aim of improving grain yield (GY) and quality.

Accurate and timely assessment of within-field GY and grain protein content (GPC) variations over the crop cycle generates different degrees of potential for N and water management/availability as indicated by Reference [2] and Reference [3], and also as a strategy for selective harvesting [4–7]. GY and GPC are two major factors for wheat production where the latter is an important determinant of the end-use value [8]. GPC is a function of the conversion of grain nitrogen (N) content into protein (further reading in References [9,10]), which is dependent on genotype and strongly influenced by environmental variables, such as timing and amount of nitrogen application, water access and temperature, especially during the grain filling period [11–15]. These factors influence the rate and duration of wheat grain development, protein accumulation and starch deposition [16–18]. The most influential environmental factor on wheat quality is the availability of soil N, which in turn is influenced by N fertilization. Therefore, proper management of N fertilizer is essential to ensure high quality wheat production.

The design of fertilizer application regimes should combine different factors (e.g., rate, placement, timing and splitting) with a view to optimize both wheat GY and GPC [19]. For example, the application of N fertilizer around heading increases GPC without reducing GY [20,21]. Hence, it is important to link N fertilization with N uptake by the plant.

The highest N uptake begins around jointing and reaches a plateau at heading. N uptake, N accumulation and the further partitioning of N within the plant are all important processes that determine GY and GPC [22,23]. N uptake, which has major influence on a crop’s green canopy and on the way N controls canopy growth and senescence, depends upon the stage of crop development—before, during and after stem extension. Canopy size determines the proportion of sunlight intercepted, and is directly related to dry matter and green biomass, with the latter shown to be strongly related to GY [24]. Differences in N uptake between the pre- and post-anthesis periods may affect N partitioning at the plant level [21] and N content in the grain as this originates from two different sources: partitioning from vegetative organs of N assimilated at pre-anthesis stages, and N uptake from the soil post-anthesis. It is understood that N accumulated before anthesis is the major source of grain N. In wheat, between 50–95% of the N content in the grain comes from the partitioning of N stored in shoots and roots before anthesis, with the leaves and stems being the most important sources of N for grain [25].

Crop nitrogen status has been widely reported to be linked to canopy reflectance because of its relation to chlorophyll content (Chlorophyll a and b; C_a+b_) [3,26,27]. Remote sensing technologies have become less expensive in recent years, and this has improved their potential for operational purposes. Remote sensing-based approaches to vegetation monitoring use two main methods to estimate canopy biophysical and biochemical traits. The first is based on parameter retrievals through vegetation canopy reflectance modeling, while the second approach focuses on developing empirical relationships between remotely sensed canopy reflectance and biophysical variables.

The second approach, which is our focus, seeks to establish empirical relationships between plant biophysical variables and vegetation indices (VIs). VIs are essentially a way of combining multispectral observations in a single metric, aiming to minimize the effect of external factors on spectral data and to derive canopy characteristics [28], whereas these external factors influencing the reflectance values may come from soil brightness, soil color, atmospheric effects, sensor calibration and differences in the spectral responses of sensors to bidirectional effects [29].

A large number of VIs have been developed to minimize those external effects to better convey information about vegetation canopy [29,30]. Indices based on both ratios and normalization formulas are referred to as Ratio Spectral Indices (RSIs) and Normalized Difference Spectral Indices (NDSIs), of which many variants have been developed that combine wavelengths of different parts of the electromagnetic spectrum (EMS).

Chlorophyll related VIs have also been developed due its relation to crop N, as previously mentioned. Combinations of VIs, such as Modified Chlorophyll Absorption in Reflectance Index (MCARI) divided by Optimized Soil-Adjusted Vegetation Index (OSAVI), have shown good results for describing leaf chlorophyll concentration [31]. Also, more pigment specific VIs such as Pigment Specific Normalized Difference c (PSNDc), Pigment Specific Simple Ratio for chlorophyll a and b (PSSRa; PSSRb), and carotenoids (PSSRc) [32] have shown to be indicators of crop N status. Based on this, canopy reflectance at specific crop stages may be a proxy for GY and GPC and may be indirectly measured using remote sensing technologies.

There is spatial variability of GY and GPC within a crop field due to soil spatial variability, micro-climate conditions and crop management [4,33–35]. Crop yield assessment using remote sensing has been considered useful [36–40]; however, it is well documented that some normalized indices, such as the Normalized Difference Vegetation Index (NDVI) [41], saturate at high leaf area index (LAI) values and are also affected by other factors, such as soil background, canopy shadows, illumination, atmospheric conditions and variation in leaf chlorophyll concentration [28,42,43]. For GPC, there are only a few studies relating it with satellite imagery [2,3,44–46] or low-altitude aerial and terrestrial proximal sensing [2,27,47–49].

The majority of those studies are based on multispectral broad-band signals on experimental designs or at the regional scale. There is usually wider range variation of the response variable at the regional scale than at the field scale [50]. In several studies, this wide range was induced, for example, in the case of GPC with different N application levels [2,3,27,46,47,49]. To the best of our knowledge, in the literature there is no detailed description of in-situ GPC data estimated through remote sensing imagery. There are also only a few studies that have explored how multi-temporal information can enhance the assessment of GY and/or GPC at a regional scale using broad-band multispectral imagery [46,49,51,52].

New methods that use hyperspectral remote sensing promote further spectral exploration of the signal, allowing the calculation of several other narrow-band VIs, suggested as potentially useful for precision agriculture [42,53]. Besides the VIs currently presented in the literature, hyperspectral signals can be used to calculate complete combinations of all available wavelengths using generic formulas, e.g., NDSI and RSI [54–59]. Exploiting the hyperspectral signal, multi-temporal spectral exploratory analysis offers new potential for assessing within-field information, and identifying the most suitable combinations of spectral wavelengths and the optimal time of image acquisition for specific precision agriculture applications.

The present study analyses the respective contribution of the spectral, narrow-band physiological vegetative indices and temporal dimensions of the hyperspectral signal, to the assessment of the within-field spatial variability of GY and GPC in two commercial wheat fields. The feasibility of this study relies on a multi-temporal and spectral exploratory analysis.

## 2 Materials and Methods

### 2.1 Field Site and Data Collection

**Figure 1:**
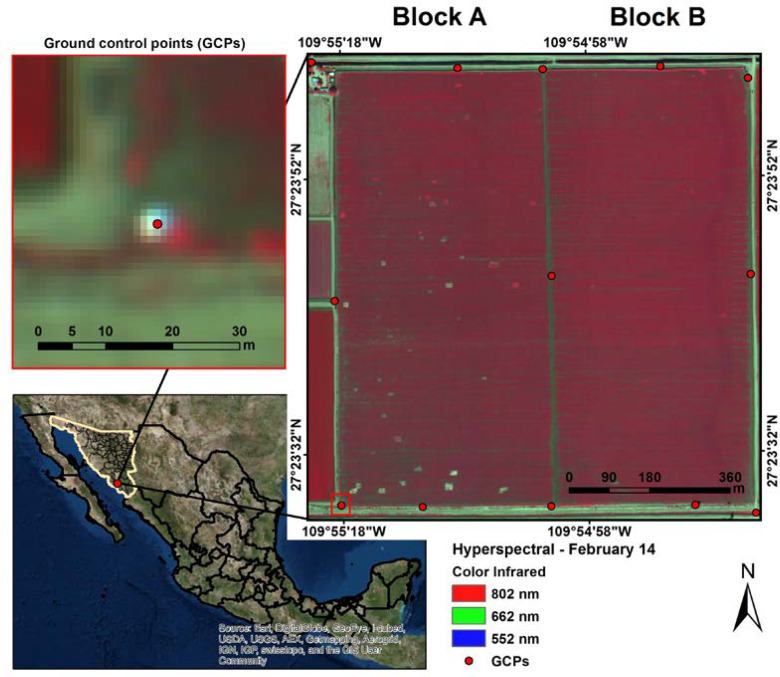
Location of the study area in Mexico, showing a hyperspectral early-season colour infrared (CIR) image (February 14) of the study fields (Block A and B) and used ground control points (GPCs).

This study was carried out in a wheat field in the Yaqui Valley near Ciudad Obregón (Sonora), in northwestern Mexico (27°23′43.83”N and 109°55″0.90”W), during the 2014 wheat crop cycle. It consisted of two furrow irrigated 43-ha blocks (called ‘A’ and ‘B’) (Figure 1). The blocks were sown with the variety Cirno-C2008 in January 2014 and harvested at the end of May 2014. The climate in the Yaqui Valley is semi-arid (Köppen climate classification subtype “Bsh”) with variable precipitation rates averaging 280 mm per year and an average daily temperature of 24*^ ◦^*C. The soils in this region are coarse sandy-clay, mixed with montmorillonitic clay. The two major soil types in the Yaqui Valley are clayey and alluvial soils at a 3:2 ratio [60].

A weekly/biweekly flight campaign took place starting from stem elongation (GS31; February 14; [61]) until just prior to harvest (GS92; grain hard, 7 May), resulting in 10 airborne mosaics (Table 1). They were acquired with a push-broom micro-hyperspectral imaging sensor (Micro-Hyperspec VNIR model, Headwall Photonics, Bolton, MA, USA) (spectral region: 400–850 nm; 250 channels) flying at 1200 m above ground in a manned airplane, yielding a ground sampling distance of circa 1 m.

**Table 1:** Image dates and respective crop growth stages.

Image Date	Crop Growth Stage
14 February	Initiation of stem elongation (GS31)
19 February	Stem elongation period
27 February	Stem elongation period
11 March	Booting (GS41)
17 March	Heading (GS55) 28 March Anthesis (GS65)
7 April	Grain filling (GS71)
15 April	Late milk (GS77)
25 April	Physiological maturity (GS87)
7 May	Grain hard (GS92)

Ground control points (GCPs) were made using 3 × 3 m long × 1 m thick crosses of polyethylene sheet coated with aluminum foil fixed with duct tape and were placed around the study sites (Figure 1) and georeferenced with a global navigation satellite system (GNSS) receiver using the real-time kinematic (RTK) technique (Trimble R4 GNSS system, Trimble, Sunnyvale, CA, USA) to ensure accuracy. Georeference processing of each image was done ensuring root mean square errors (RMSE) lower than the image resolution.

The micro-hyperspectral instrument was radiometrically calibrated in the laboratory using derived coefficients with a calibrated uniform light source (integrating sphere, CSTM-USS-2000C Uniform Source System, LabSphere, North Sutton, NH, USA) at four levels of illumination and six integration times. Hyperspectral imagery was atmospherically corrected using the total incoming irradiance at 1 nm intervals simulated with the SMARTS model developed by the National Renewable Energy Laboratory, US Department of Energy [62,63]. Therefore, the aerosol optical depth was measured at 550 nm with a Micro-Tops II sun photometer (Solar LIGHT Co., Philadelphia, PA, USA) in the study area at the time of the flights. SMARTS computes clear sky spectral irradiance, including direct beam, circumsolar, hemispherical diffuse, and total irradiance on a tilted or horizontal plane for specified atmospheric conditions. The algorithms were developed to match the output from the MODTRAN complex band models to within 2%, using aerosol optical depth as an input. The spectral resolution was 0.5 nm for the 280–400 nm and 1 nm for the 400–1750 nm ranges of the electromagnetic spectrum. This radiative transfer model has been previously used in other studies [64–68] for the atmospheric correction of narrow-band multispectral imagery.

Manual grain sampling [69] took place just before harvest using a half regular and a half stratified grid of 50 sampling points in Block B (100 points in total). Reallocation of 10% of the regular grid points to shorter distances than the original grid was done to minimize the ratio of the smallest to largest sample distance [70,71]. This aimed to define the range of spatial dependence [72] of the response variables. In Block A, 14 sampling points were selected based on a visual inspection of the soil apparent electrical conductivity (EC_a_) map [73], to cover the full EC_a_ range variation. The decision for different number of points in each block was due to budget constraints and to have at least a reasonable number of points (100) in one of the fields to capture its spatial variability and enable mapping using variography [74]. Each sampling point was based ona2m^2^ frame centered on the point geocoordinates, where all wheat plants were harvested, threshed and GY measured. A grain sub-sample was taken for laboratory quality analysis, where GPC (%) and moisture content (%) were determined by NIR spectroscopy (NIR Systems 6500, Foss, Hilleroed, Denmark) calibrated according to official AACC standard methods 39–10 and 46–11A [75]. The GY and GPC values were reported at 12.5% moisture basis. Descriptive statistics of the sample were computed using the corresponding variables.

Spectral binning was performed on each mosaic into 7.5 nm FWHM (Full Width at Half Maximum) to decrease noise effects, resulting in 62 wavelengths. From those, the 751, 759, 766, and 773 nm wavelengths were removed due to oxygen absorption by the sensor. Finally, 58 wavelengths were used for subsequent analyses. Spectral information was extracted from each image using the point sampling location from both blocks (*n* = 114). The extraction was done by taking the average of a 3 × 3-pixel window (9 m^2^) around each sampling point to account for minor shift of the images during orthomosaic processing. The resulting averaged spectral data set was used for the following statistical and remote sensing analyses regarding GY and GPC.

### 2.2 In Situ Data Description—GY and GPC Descriptive Statistics, Correlation Analysis and Hyperspectral Profiles

The GY and GPC data from Block B were interpolated onto a 3-m grid (pixels of 9 m^2^) using global block kriging, fitting the best global variogram according to the spatial variability of the data in VESPER [76]. The purpose of this mapping was to visualize the within-field spatial variability of the response variables. Map displays were done using the ArcGIS software suite (v10.1; ESRI, Redlands, CA, USA).

The relationship between GY and GPC was analyzed using Pearson’s correlation coefficient. As stated by many authors [4,21,77–80], GY is usually negatively correlated to GPC. However, [80] demonstrated that this correlation may vary from negative to positive within a farmer’s field due to soil spatial variability and crop management interactions. Based on this context, we decided to further explore the within-field relationship of GY and GPC by applying a moving window approach to check the spatial local correlation between both variables. The moving window approach was applied only to block B due to its denser data sampling (100 sampled points). It consisted of a 150 × 150 m window which moved across the block collecting the neighbors within this window, using as basis the same 100 sampled point locations. The window dimension was chosen to respect an average of 10 degrees of freedom for the correlation analysis. Correlation results were considered significant if *p* < 0.05.

The hyperspectral profiles of the highest and lowest GY and GPC sampling points only were extracted for each image and plotted on a single graph for each response variable. The choice to limit the graph to these two responses, and the decision not to plot the mean or median response, was made to visualize the possible reflectance differences for both levels (highest and lowest) of the response variables without generating an overcrowded and unintelligible graph.

Two exploratory analysis approaches were selected for data analysis of the acquired hyperspectral imagery time series: (a) a spectral approach; and (b) a multi-temporal approach. The spectral exploratory analysis approach is based on a complete two by two combination of all available wavelengths into NDSI and RSI spectral indices (SI). For the multi-temporal exploratory analysis approach, two methods were tested: temporal principal component analysis (tPCA) and the integration of VIs over time using an area under the curve (AUC) approach.

### 2.3 Spectral Exploratory Analysis Using Narrow-Band Physiological Spectral Indices

For the spectral exploratory analysis, we applied two types of generic formulas to generate SIs. Using complete two by two combinations of spectral wavelengths, the ratio spectral index (RSI) and the normalized difference spectral index (NDSI) were calculated. The RSI (Equation (1)) and NDSI (Equation (2)) are defined as:

1 $$CSI=\frac{\left(GA-GGA\right)}{GA}\times 100$$

2 $$CSI=\frac{\left(GA-GGA\right)}{GA}\times 100$$

where *Ri* and *Rj* are the reflectance for wavelengths *i* and *j*, respectively. Representations of RSI and NDSI are the PSSRa and NDVI, whereby both indices use NIR and R wavelengths. Spectral indices based on the complete two by two combinations of the hyperspectral signal were generated by applying the RSI and NDSI formulas, similar to studies by [54–59]. Subsequently, regression analyses were performed using RSI and NDSI indices as predictors for all GY and GPC data points. Contour maps of the coefficient of determination (R^2^) describing the relationships of these SIs with GY and GPC were generated. These maps provide inclusive information on the optimum pair of wavelengths to assess both response variables all along the crop cycle [54,55].

### 2.4 Multi-Temporal Spectral Exploratory Analysis

#### 2.4.1 Temporal Principal Component Analysis (tPCA)

The relatively high temporal resolution flight campaign (here: 10 hyperspectral mosaics within one crop cycle—February to May) allowed for a multi-temporal approach to analyze the importance of the mosaics and wavelengths all along the crop cycle and at specific phenological stages (crop growth stages). For this purpose, a standardized principal component analysis (PCA) was applied to each individual wavelength across the 10 images, with 10 principal components (PCs) recorded for each wavelength. The first two PCs were considered in a Pearson’s correlation analysis with GY and GPC. The eigenvector and eigenvalue matrices were assessed to determine which image and wavelength across the crop cycle carried the highest proportion of variance within the dataset.

#### 2.4.2 Integration of VIs Over Time

For further data exploration, four specific VIs for each response variable were chosen according to their potential to deliver structural and chlorophyll information (Table 2). Also, the relationship of VIs with GY and GPC was a selection criterion, as presented in preliminary results by [73,81]. Both canopy structure and its greenness are indicators of the N-status of the plants, where the leaves and stems are stated to be the most important sources of N for the grain [25].

**Table 2:** Selected VIs for GY and GPC.

Index	Formula	Reference
**GY**		
Enhanced vegetation index (EVI)	$2.5*\left(\frac{R_{800}-R_{770}}{R_{800}+6*R_{670}-7.5*R_{400}+1}\right)$	[82]
Modified triangular vegetation index 2# (MTVI2)	$\frac{1.2*\left[1.2*(R_{800}-R_{550})-2.5*\left(R_{670}+R_{550}\right)\right]}{\sqrt{{\left(2*R_{800}+1\right)}^{2}-(6*R_{800}-5*\sqrt{R_{670}})-0.5}}$	[83]
Normalized difference vegetation index (NDVI)	$\frac{R_{800}-R_{680}}{R_{800}+R_{680}}$	[41]
Optimized soil-adjusted vegetation index (OSAVI)	$\frac{(1+0.16)*\left(R_{800}-R_{670}\right)}{\left(R_{800}+R_{670}+0.16\right)}$	[84]
**GPC**		
Ratio: Modified chlorophyll absorption ratio index/Optimized soil-adjusted vegetation index (MCARI/OSAVI)	$\frac{\left[\left(R_{700}-R_{670}\right)-0.2*\left(R_{700}-R_{550}\right)\right]*\frac{R_{700}}{R_{670}}}{\left(1+0.16\right)*\left(R_{800}-R_{670}\right)/\left(R_{800}+R_{670}+0.16\right)}$	[31]
Pigment specific normalized difference c (PSNDc)	$\frac{R_{800}-R_{470}}{R_{800}+R_{470}}$	[32]
Pigment specific simple ratio for carotenoids (PSSRc)	$\frac{R_{800}}{R_{470}}$	[32]
Transformed chlorophyll absorption in reflectance index (TCARI)	$3*\left[\left(R_{700}-R_{670}\right)-0.2*\left(R_{700}-R_{550}\right)\right]*\frac{R_{700}}{R_{670}}$	[85]

The VIs’ temporal profiles based on the highest, median and lowest GY and GPC points within the field were plotted. Based on this information, the area under the curve (AUC) using the trapezoidal method (Riemann’s integrals) was calculated for each selected VI on each sampling point observation, aiming to integrate its temporal information across the crop cycle for different crop growth stages (Table 3).

**Table 3:** Range of crop growth stages used to calculate the area under the curve (AUC) for different vegetation indices.

Code	Description of Crop Growth Stage	Dates of Used Images	Number of Mosaics
AUC1	Whole time series	14 February to 7 May	10
AUC2	Steam elongation (GS31) to booting (GS41)	14 February to 11 March	4
AUC3	Booting (GS41) to anthesis (GS65)	11 March to 28 March	3
AUC4	Heading (GS55) to anthesis (GS65)	17 March to 28 March	2
AUC5	Steam elongation (GS31) to anthesis (GS65)	14 February to 28 March	6
AUC6	Grain filling (GS71) to late milk (GS77)	7 April to 15 April	2
AUC7	Grain filling (GS71) to physiological maturity (GS87)	7 April to 25 April	3
AUC8	Grain filling (GS71) to grain hard (GS92)	7 April to 7 May	4

The AUC for each VI and crop growth stage were calculated and the area regressed against GY and GPC using linear regression (software R: package “plyr” for AUC and package “stats” for regression analysis; [86,87]). Regression results were considered significant if *p* < 0.05.

### 3 Results and Discussion

#### 3.1 In Situ Data Description—GY and GPC Descriptive Statistics, Correlation Analysis and Hyperspectral Profiles

Both GY and GPC showed a distribution with mean and median values close to each other. This reflects a broadly symmetrical (normal) distribution although with a high value of kurtosis for GPC (Table 4). The GPC data are concentrated between 12.03% (1st Quartile) and 12.56% (3rd Quartile) with the mean of 12.32%. Relatively low GPC ranges might occur for irrigated fields under natural conditions and conventional agricultural practices, as N and water are applied equally and at high quantities throughout the field and soil spatial variability may have a limited effect on canopy variability. However, higher GPC ranges have been reported in rain-fed crop management fields, where soil spatial variability strongly influences water and N availability [80].

**Table 4:** Descriptive statistics for GY and GPC from both blocks (*n* = 114).

	GY	GPC
Maximum	8.02	14.96 3º
Quartile	6.84	12.56
Median	6.36	12.25
1º Quartile	5.98	12.03
Minimum	4.66	10.87
Mean	6.41	12.32
Skewness	0.02	1.25
Kurtosis	−0.14	5.67
CV	11.51	4.15

GY—Mg ha^−1^; GPC—%.

**Figure 2:**
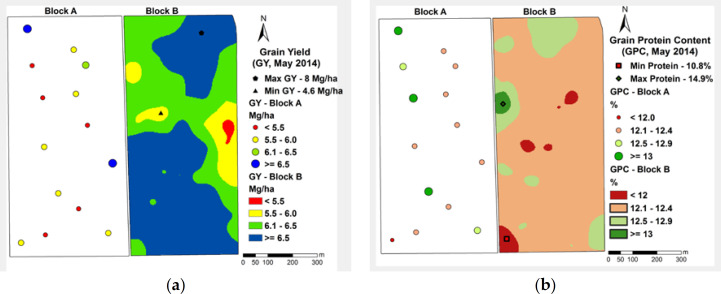
(**a**) GY and (**b**) GPC maps based on manually-collected grain samples at the two study sites (Block A: only point values, *n* = 14; Block B: spatially interpolated point values using block kriging, *n* = 100) (GPC-threshold = 12.5% according to the farmer’s recommendation).

Through the mapping exercise (Figure 2), it is possible to visualize the within-field spatial variability for GY and GPC, which ranged from around 4.6 to 8 Mg ha*^−1^* and 11 to almost 15%, respectively. The threshold of 12.5% for GPC was chosen based on the farmer’s recommendation to differentiate between low and high wheat quality based on premium quality and better market prices. Although the maximum value of 14.96% for GPC may be considered an outlier, after a few analyses (data not shown), we decided to keep the data point. There was no further evidence this value was an error of measurement. GPCs of around 14–15% within farmers’ fields and on a regional scale are reported throughout the literature [4,45,80]. The highest and lowest GY and GPC points were identified among the sampled data points and maps, and afterwards used as references for extracting the reflectances from the images during the crop cycle.

GY and GPC did not show significant correlation (r = −0.09; *p*-value = 0.49) when we used all data points from the entire field. However, local spatial variation in the correlation between GY and GPC obtained through the moving window approach showed correlation coefficients varying from −0.76 to +0.61. Just two negative coefficients were significant at 5% probability (−0.76 and −0.69) and one positive (0.61) at 10% probability among the other 97 coefficients (Table 5).

**Table 5:** Descriptive statistics of Pearson’s correlation coefficients between GY and GPC obtained through the moving window process.

	r Coefficient	Respective p-Value	Respective Degrees of Freedom
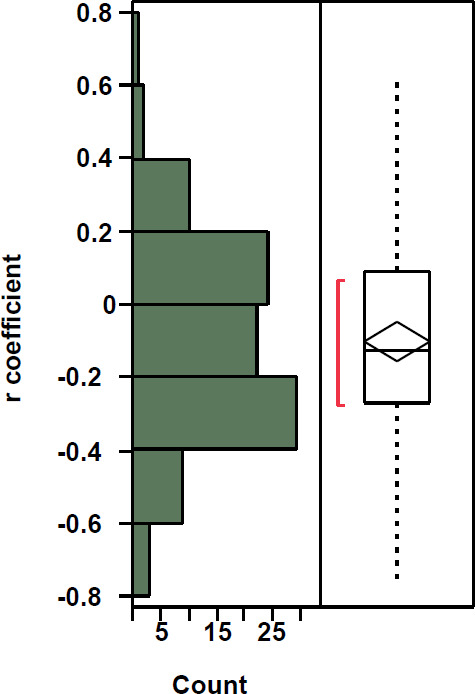	Maximum	0.61	0.07	7
	3º Quartile	0.08	0.80	9
	Median	−0.12	0.71	9
	1º Quartile	−0.26	0.30	15
	Minimum	−0.76	0.04	5
	Mean	−0.10	0.35	9
	Skewness	0.10	-	-
	Ku rtosis	-0.04	-	-
	n	100	-	-

These results agree with results obtained by Reference [80], where GY and GPC data (acquired from on-the-go sensors mounted on harvesters in 27 fields over 3 seasons) were evaluated. The majority of these fields showed bigger areas of negative local correlations for the first two seasons and positive for the third season; however, a considerable percentage of the area had non-significant coefficients for all seasons/fields.

Areas within the field where GY and GPC were negatively correlated could represent conditions where effective access to N has been relatively uniform, but with limited access to water due to soil spatial variability structure, which limited grain growth. Areas with positive coefficients could represent more access to water but limited N availability [80]. Soil apparent electrical conductivity (EC_a_), which may be a proxy for plant available soil water storage capacity (PAWC, [88]), was quite variable in this field [73]. This supports the within-field spatial variation of the correlation between GY and GPC and reinforces the need for exploring both variables separately.

**Figure 3:**
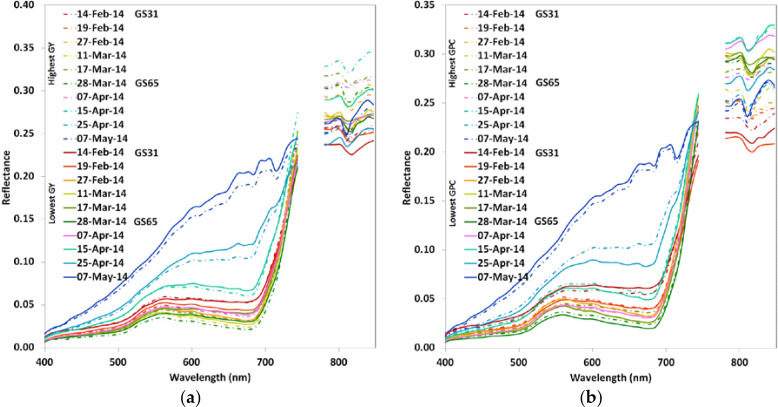
Reflectance profile from the highest and lowest GY (**a**) and GPC (**b**) measured sampling points across blocks.

The previously identified highest and lowest GY and GPC locations (Figure 2a,b) were used to extract the hyperspectral signal from the mosaics. The reflectance profile of the highest (dashed lines) and lowest (full lines) GY region is represented in Figure 3a. The dark red dashed and full lines (14 February) represent the GS31 stage, which is critical, as it is a stage where N is often applied and an early stage where nutrient stress diagnosis in wheat may be made. The dark green (dashed and full) lines represent anthesis (28 March), supposedly the peak of vegetative development. At GS31, a small reflectance difference in the NIR region is detectable. This difference increases up to 15 April, and decreases at physiological maturity (GS87, 25 April) and grain hard (GS92, 7 May).

The reflectance profile (Figure 3b) from the highest (dashed lines) and lowest (full lines) GPC region showed similar spectral behavior from 400 to 770 nm, except for the mosaic from 25 April (end of grain filling stage), which showed some difference in reflectance in the R region (around 680 nm). At 840 nm (NIR), it showed some differences in the reflectance for the highest and lowest GPC region.

Zarco-Tejada et al. [42] found similar reflectance behavior for low and high growth areas of cotton. In the case of wheat, the best time to diagnose for GY is at the beginning of stem elongation (GS31) and for GPC around heading/anthesis stage, as that is when GPC can be increased through nitrogen management [20]. Therefore, identifying differences in reflectance at an early stage (GS31) and at heading/anthesis makes it possible to diagnose nitrogen stress while there is still time to raise GY and GPC through crop management.

### 3.2 Spectral Exploratory Analysis Using Narrow-Band Physiological Spectral Indices

**Figure 4:**
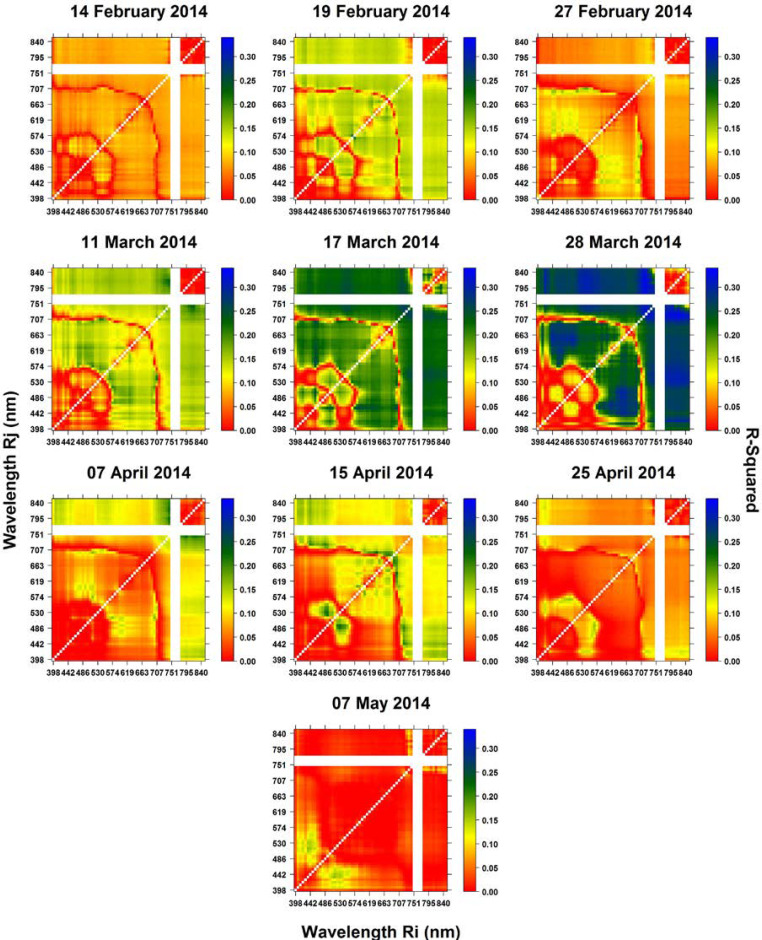
Contour maps of the coefficient of determination (R^2^) between NDSI (*Ri, Rj*) and GY using the complete combinations of two wavelengths at *i* and *j* nm.

**Figure 5:**
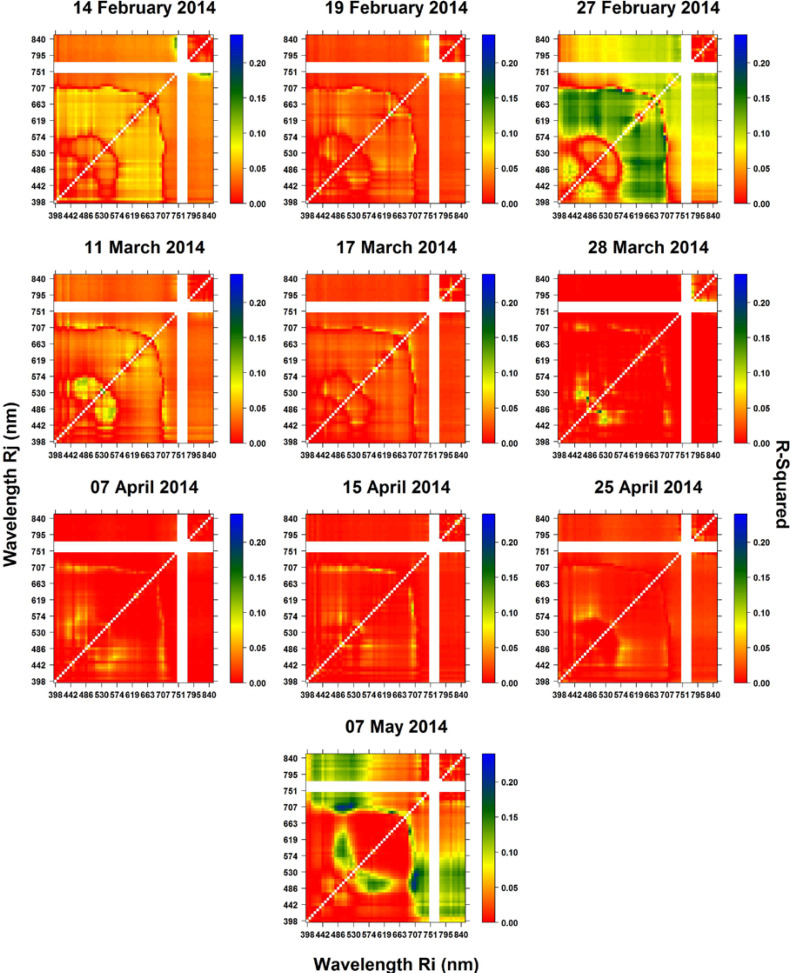
Contour maps of the coefficient of determination (R^2^) between NDSI (*Ri, Rj*) and GPC using the complete combinations of two wavelengths at *i* and *j* nm.

Since the results of the regression analysis using RSI and NDSI were very similar, only NDSI results are shown here. The contour maps of the coefficients of determination (R^2^) are shown in Figures 4 and 5, which are the results of the regression analyses of the NDSI indices versus GY and GPC, respectively.

Using the R^2^ contour maps, it is possible to infer which wavelength combination performed better when assessing GY and GPC (Figures 4 and 5). The relationship between crop yield and VIs has been widely studied in the literature. Many authors have reported good correlations between the yield of major commodity crops such as wheat, maize, and cotton based on multispectral broadband data imagery and mostly using combinations of NIR, R and green (G) spectral regions [36–40,89,90]. In the current study, mosaics around booting and anthesis led to the highest relationship with GY (Figure 4). The R^2^ varied from 0.22 to 0.32 (booting to anthesis; GS41–GS65; 11–28 March). The best results across these images were with combinations between broad parts of the NIR region (*Ri*, 750 to 840 nm—except 751, 759, 766, and 773 nm which were previously removed) and narrow bands of the RE (*Rj, ±*720–736 nm) region. In these regions, reflectance is linked with biomass, which has been shown to be related to GY [91–93]. These results are followed by combinations of broad parts of NIR and the visible region of the spectrum. Here, all wavelengths between 400 and 680 nm (*Rj*) combined with NIR obtained slightly lower adjustments than NIR and RE combinations with peaks at NIR (*Ri*) and blue (B)/G regions (*Rj*). Similar results were highlighted in the studies by [30,94].

As well as revealing combinations between wavelengths in the NIR, RE and visible spectral regions, relationships between combinations solely within the visible spectrum were also detected. Although with a lower R^2^, G/R regions (*Ri*) combined with B (*Rj*) showed consistent relationships throughout the three images acquired from booting to anthesis. The correlations between GY and the combinations within the visible spectrum are supported by the fact that chlorophyll (C_a+b_) has two peaks of light absorption in the B and R regions [95]. Also, carotenoids display peak absorption in the B region [96].

GPC is a function of the conversion of plant nitrogen content into protein, so it is expected that plant nitrogen concentration estimated through remote sensing techniques may be able to partially explain GPC variation [45]. A few studies have reported the potential use of individual wavelengths and/or different VIs such as PPR ([R550-R450]/[R550 + R450]), NDVI ([R810-R680]/[R810 + R680]), RVI (R810/R680), GNDVI ([R810-R560]/[R810 + R560]) and GRVI (R810/R560) to describe nitrogen status and GPC [3,27,45–47,49]. All of the former studies have used VIs derived from G, R and NIR wavelengths through normalized and simple ratio formulas. In our study, NDVI, for instance, was not one of the top 10 coefficients with GPC across the images and growth stages. However, most previous studies were carried out under controlled conditions with low and high levels of nitrogen application (plot or trial design). In contrast, this study was carried out in an irrigated farmer’s field without nitrogen level treatments (respectively under natural conditions), resulting in a low range of GPC variability (12.03–12.56% from the 1st to 3rd Quartile; Table 4). This may explain the rather low coefficients of determination (R^2^ *≤* 0.21) obtained in the results.

Through the R^2^-contour maps (Figure 5), it is also possible to visualize the wavelength region where R^2^ was predominantly high, such as from 574 to 700 nm on *Ri* and 400 to 574 nm on *Rj* at pre-booting (27 February), and 707 to 840 nm on *Ri* and 486 to 530 nm on *Rj* after physiological maturity (7 May). All dates show slight R^2^ differences within those regions of the spectrum. The highest R^2^ within the images from 27 February (pre-booting; GS41) (R^2^: 0.20) and 7 May (grain hard; GS92) (R^2^: 0.21) were obtained with narrow band combinations of the RE and G regions (27 February: 700, 574 nm; 7 May: 707, 523 nm). At booting, plants are increasing N uptake, and physiological maturity is the end of grain filling period, where N is redistributed from photosynthetic tissues to form GPC [97]. Although the complete two by two combinations of wavelengths provided improved SIs for the assessment of GPC against more traditional VIs, the results indicate that the visible near-infrared (VNIR) portion of the electromagnetic spectrum may not have enough potential to assess GPC at the field level. The shortwave infrared (SWIR) portion of the electromagnetic spectrum, where water content is the main determinant of leaf spectral behavior [98], may be of use for this specific trait. For technology development, the obtained information should support the wavelength decision for sensor development aimed at measuring GPC; however, different environmental factors, such as drought and heat, may also need to be taken into consideration.

In summary, the complete two by two combinations of spectral wavelengths (presented as R^2^-contour maps) proved to be a simple and robust method for exploring hyperspectral data. Here, combining NIR and RE spectral wavelengths into a normalized formula was shown to be more suitable for assessing GY and combinations of RE and G for assessing GPC. For the assessment of GY and GPC, better results were achieved with images acquired during anthesis (GY) and around booting and just after physiological maturity (GPC).

### 3.3 Multi-Temporal Spectral Analysis

#### 3.3.1 Temporal Principal Component Analysis

**Figure 6:**
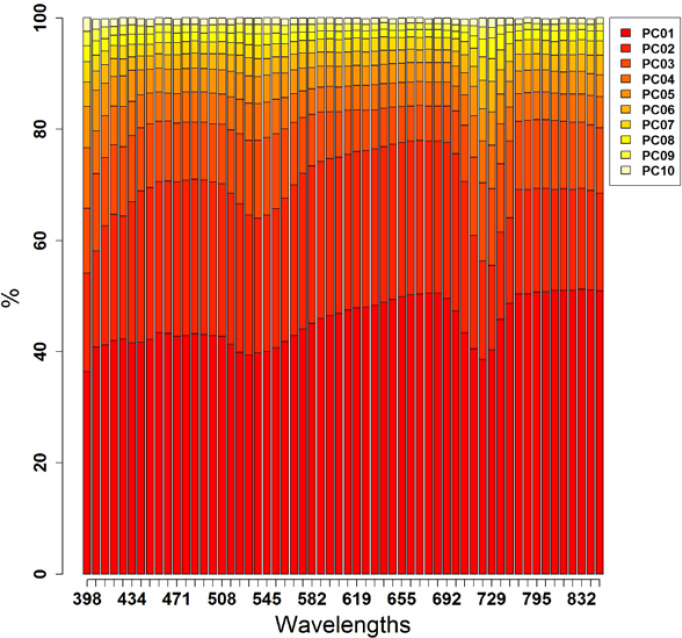
Eigenvalues/percentage of variance of each PC within each wavelength.

The contribution to the total variance of each obtained PC for each wavelength is shown in Figure 6. Across all wavelengths, all PCs followed a similar course of fluctuation with (a) no data noise, (b) lower percentages at wavelengths in the violet (V) (~400 nm), G (~530 nm), and red-edge (RE) (~720 nm) regions, and (c) higher percentages at wavelengths in the B (~485 nm), R (~685 nm), and NIR (~830 nm) regions of the electromagnetic spectrum. Nevertheless, as the first PCs contribute more than the last PCs, generally most data noise is stored in the last PCs (here: PC6 to PC10) due to the PCA transformation process. PC1 (40–50% of the variation in each wavelength, except in some wavelengths in the V, G and RE regions) and PC2 (>25%, except in some wavelengths in the V, G and RE regions as well) explained the majority of the variation in each wavelength. PC1 made its highest contribution in wavelengths in the R and NIR region, and PC2 just in the R region. Together, >75% was obtained in wavelengths between 610 nm and 700 nm (R region). According to these results and taking into consideration Kaiser’s criterion [99] for PC selection (eigenvalue > 1), PC1 and PC2 of each wavelength were selected for subsequent statistical analyses.

**Figure 7:**
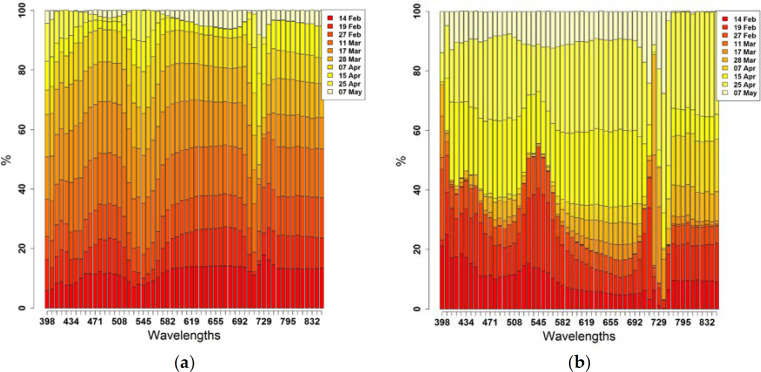
Percent contribution of each image to the total variance, per wavelength, for PC1 (**a**) and PC2 (**b**).

To determine which mosaic across the crop cycle carries the highest proportion of variance within the dataset, the eigenvector and eigenvalue matrices were computed. For PC1 and PC2, Figure 7a,b depicts the percentage contribution to the total variance per wavelength of each image.

For PC1 (Figure 7a), all images (except 25 April and 7 May) followed the previously described course of fluctuation across all wavelengths with peaks in the B, R and NIR regions and depressions in the V, G and RE regions. Their contributions range almost equally between ~10 and 18%. Continuously over almost all wavelengths, the highest percentages (~18%) were obtained from images from 11 March (booting; GS41) and 17 March (heading; GS55), followed by images from 14 February (stem elongation; GS31) to 27 February (pre-booting; GS41), 28 March (anthesis; GS65) and 7 April (grain filling; GS71) (~10 to 15%). Very low percentages (~5%) were obtained from images taken on 15 April (late milk; GS77) to 7 May (grain hard, GS92), with the exception of the RE and NIR regions from the image of April 15 (~10%). The B and R spectral region is known for having absorbance peaks for C_a+b_. NIR has high reflectance values from healthy vegetative organs with strong internal cellular geometry. At the booting stage (GS41), wheat plants are at approximately 40% of maximum growth and N uptake increases considerably. Meanwhile, the heading stage (GS55) comes just before the plant reaches its maximum green area index, which influences canopy reflectance and N uptake and reveals the importance of both stages in crop development [97].

In contrast to PC1, Figure 7b reveals that—based on PC2—late-season images between 15 April (late milk; GS77) and 25 April (physiological maturity; GS87) have a very high proportion of variance (~20 to 30%) across almost all wavelengths (except the NIR region of 15 April). Also, the formerly (based on PC1) lower contributing images from 14 February (stem elongation; GS31) to 27 February (pre-booting; GS41) show high percentages with peaks in the V, G and RE regions (~10 to 20%). Lower percentages (<10%) were found from images acquired between 11 March (booting; GS41) and 7 April (grain filling; GS71) across almost all wavelengths, except the RE (~720 nm) and NIR (~830 nm) regions. At the late milk stage (GS77), senescence and the rapid redistribution of soluble reserves have begun, which defines grain size and weight, while after physiological maturity (GS87), the grain will continue to lose moisture until it is ready for harvest.

**Figure 8:**
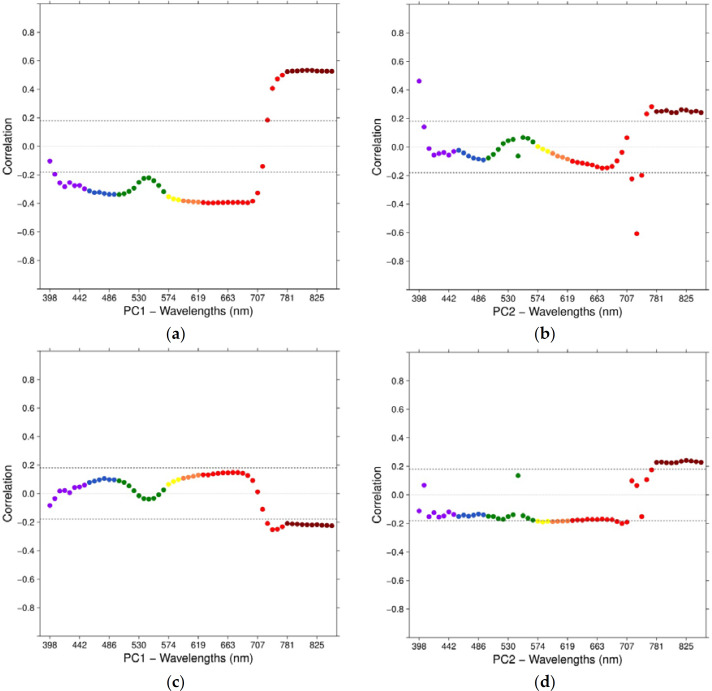
Correlation coefficients between each wavelength from PC1 with GY and GPC (**a**,**c**) and wavelengths from PC2 with GY and GPC (**b**,**d**). The dashed line represents a 5% significance level.

Pearson’s correlation coefficient was calculated to understand the relationships of each wavelength from the selected PCs with GY and GPC, respectively. With GY, PC1 had weakly significant (−0.4 *≤* r *≤ −*0.3) correlations across almost all wavelengths from the B to the R region, except in the G region. Moderately significant correlation coefficients (around +0.5) were found mainly for the NIR wavelengths (Figure 8a). For PC2, there were no significant correlations (−0.2 *≤* r *≤* +0.2) across almost all wavelengths, except very weak correlations (*±*0.2 < r *≤±*0.3) with some RE and NIR wavelengths (Figure 8b). Overall, the strongest relationship was revealed with PC2 using the RE 722 nm wavelength (−0.6), where images from 17 March (GS55, heading) and 28 March (GS65, anthesis) revealed the highest loadings of 37% and 33%, respectively.

With GPC, PC1 as well as PC2 correlated weakly (*±*0.2 < r *≤±*0.25) using some RE and all NIR wavelengths, with the 729 nm wavelength from PC1 having the highest coefficient (−0.25) and its surrounding wavelengths (722, 736 and 744 nm) slightly different coefficients. Images from 14 and 27 February and 11 March had the highest loadings (17%, 15% and 16%, respectively). Both PCs have in common that no correlations existed across all wavelengths from the V to the R region (Figure 8c,d).

In general, both PCs and their correlations with GY and GPC showed a smooth behavior across the available spectrum wavelengths, where peaks of correlation coefficients were shown at B, R and NIR spectral regions on PC1 for GY, with similar, although non-significant, inverted behavior for GPC. Although PC2 exhibited some noisy wavelengths within the G and RE regions for GPC, it still showed a continuous pattern across the available spectrum.

#### 3.3.2 Integration of VIs Over Time

**Figure 9:**
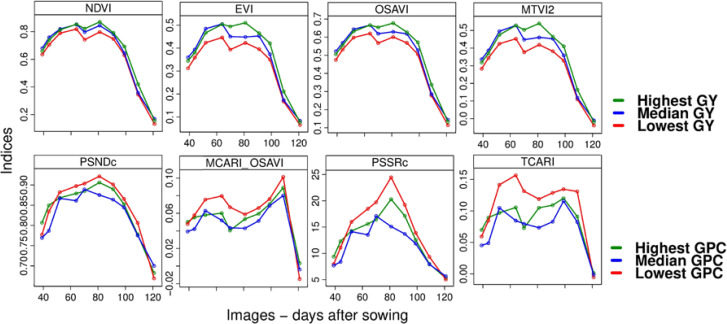
Temporal profiles of selected vegetation indices (VIs) to the within-field highest, median and lowest measured GY and GPC.

Another approach for performing a temporal exploratory analysis of a hyperspectral image time series is the integration of VIs over time through the calculation of the area under the curve (AUC). The temporal profiles for the lowest, median and highest GY and GPC within-field values are shown in Figure 9.

For GY, all VI temporal profiles revealed that the curves of highest, median and lowest GY followed a very similar course over the crop season with (a) increasing VI values during early growth stages (steam elongation to pre-booting; GS31–GS41; 14–27 February), (b) peaks at booting (GS41; 11 March) and anthesis (GS65; 28 March), (c) a slight depression at heading (GS55; 17 March), and d) a strong descending response starting from late milk (GS77; 15 April) until grain hard (GS92; 7 May). Furthermore, the highest GY values always showed higher VI values and vice versa, which is expected due to their plant structural information and its correlation to biomass and, consequently, to GY [91–93].

In contrast to GY, the VI temporal profiles for GPC show a different pattern over time for each VI. With exception of the median GPC curve, the highest and lowest GPC curves followed a similar course, whereby—starting from pre-booting crop stage (GS41; 27 February)—the lowest GPC values are always related to higher VI values. The low range variation of GPC (refer to Section 3.1) may be the reason of the ‘noisy’ effect in the VI response of the median GPC. The peaks vary: For PSNDc and PSSRc at anthesis (GS65; 28 March), for MCARI/OSAVI at physiological maturity (GS87; 25 April), and for TCARI at booting (GS41; 11 March) and late milk (GS77; 15 April). The VIs MCARI/OSAVI and TCARI indicate a higher potential for GPC estimation at late season, PSNDc and PSSRc at mid-season. Pigment-related VIs have shown to be negatively related to chlorophyll at specific crop stages [27], which is enough to explain the high VI responses to the lowest GPC value.

To understand the prediction potential of multi-temporal hyperspectral image analysis for GY and GPC, the relationships between the AUC of selected VI temporal profiles and the values of both GY and GPC were statistically evaluated at different crop stages (AUC1 to AUC8; Table 3). Table 6 summarizes the R^2^ resulting from fitting values between AUCs from selected VI profiles with GY and GPC.

Overall, independently of the crop stages (AUC1 to AUC8), the relationships across all VI temporal profiles were weak for GY (R^2^ *≤* 0.32) and very weak for GPC (R^2^ *≤* 0.1).

However, when focusing on GY, the best R^2^ were found for AUC1 using EVI and OSAVI (R^2^: 0.32), followed by AUC4 (R^2^: 0.28; PSSRc), AUC7 (R^2^: 0.27; TCARI), and AUC3 (R^2^: 0.26; PSSRc). This means that the prediction potential improved slightly (by just 4–6%) using 10 hyperspectral mosaics over the whole crop cycle (AUC1) compared to using 2 mosaics between heading and anthesis (AUC4) or 3 mosaics between grain filling and physiological maturity (AUC7) or booting and anthesis (AUC3). As such, this may not justify making such an investment for highly temporal time series of images.

The best results for GPC were obtained with the temporal VI profiles of TCARI and MCARI/OSAVI for the following AUCs: AUC5 (R^2^: 0.1; TCARI, MCARI/OSAVI), AUC2 (R^2^: 0.09; TCARI) and AUC3 (R^2^: 0.08; MCARI/OSAVI). This indicates that the most interesting period is from early to midseason, respectively from steam elongation (GS31) to anthesis (GS65). This is also the period when most N uptake (about 60%) takes place and the period of major photosynthetic capacity [100], which influences canopy expansion and, consequently, the green area index [97]. This same AUC approach was used for all NDSI combinations (data not shown). The results did not show any improvement in comparison with the single mosaic NDSI approach (spectral exploratory analysis section).

**Table 6:** R-squared of fitting between the areas under the curve (AUC) of the selected vegetation index profiles for different crop stages and GY and GPC.

	AUC1; #10		AUC2; #4		AUC3; #3		AUC4; #2		AUC5; #6		AUC6; #2		AUC7; #3		AUC8; #4	
	GY	GPC	GY	GPC	GY	GPC	GY	GPC	GY	GPC	GY	GPC	GY	GPC	GY	GPC
NDVI	**0.28**	0.01	**0.12**	**0.06**	**0.25**	0.01	**0.26**	0.01	**0.20**	**0.04**	**0.13**	0.01	**0.13**	0.01	**0.10**	0.02
EVI	**0.32**	0.02	**0.15**	**0.08**	**0.25**	0.02	**0.25**	0.01	**0.22**	**0.06**	**0.22**	-	**0.22**	0.01	**0.18**	0.01
OSAVI	**0.32**	0.01	**0.14**	**0.07**	**0.25**	0.02	**0.26**	0.01	**0.22**	**0.05**	**0.20**	-	**0.18**	0.01	**0.14**	0.02
MTVI2	**0.31**	0.02	**0.15**	**0.08**	**0.25**	0.02	0.25	0.01	**0.22**	**0.07**	**0.21**	-	**0.21**	0.01	**0.16**	0.01
PSNDc	**0.28**	0.01	**0.11**	**0.05**	**0.25**	0.01	**0.26**	0.01	**0.19**	**0.04**	**0.14**	-	**0.15**	0.01	**0.12**	0.02
MCARI/OSAVI	**0.08**	**0.06**	**0.06**	**0.06**	0.01	**0.08**	-	**0.07**	**0.05**	**0.10**	-	**0.07**	**0.09**	**0.07**	0.03	-
PSSRc	**0.24**	0.02	**0.10**	**0.06**	**0.26**	0.02	**0.28**	0.01	**0.19**	**0.05**	**0.13**	-	**0.14**	0.01	**0.13**	0.01
TCARI	**0.24**	**0.07**	**0.10**	**0.09**	**0.11**	**0.07**	**0.09**	**0.05**	**0.13**	**0.10**	**0.12**	**0.04**	**0.27**	0.01	**0.16**	-

AUCl—Whole time series of images; AUC2—images from steam elongation (GS31) to booting (GS41); AUC3—images from booting (GS41) to anthesis (GS65); AUC4—images from heading (GS55) to anthesis (GS65); AUC5—images from stem elongation (GS31) to anthesis (GS65); AUC6—images from grain filling (GS71) to late milk (GS77); AUC7—images from grain filling (GS71) to physiological maturity (GS87); AUC8—images from grain filling (GS71) to grain hard (GS92). # indicates the number of mosaics used in each AUC. Bold r—squared are significant at p < 0.05.

Other authors have made use of remote sensing data acquired throughout the season to develop spectral growth profiles based on VIs. Using NDVI as an indicator, Dubey et al. [101] found that the area under the growth profile explained nearly 69% (R^2^ around 0.47) of GY variability in wheat production districts in Indian states. Similarly, Xue et al. [49] analyzed cumulative VIs for diverse crop growth periods (jointing to maturity, booting to maturity, heading to maturity) to estimate GPC. However, in our case, the use of accumulated VIs showed no significant improvement in the correlations with GPC compared to single VIs.

In summary, multi—temporal spectral exploratory analysis showed its potential for identifying the optimal image timing across the crop cycle and reflectance wavelengths. Temporal PCA revealed that B, R and NIR spectral information at booting and heading crop stages carried the highest variance of the dataset, followed by late season images across almost all wavelengths, except NIR. Moreover, this approach showed the smooth behavior of the spectrum across the crop cycle, where peaks of correlation coefficients occurred at B, R and NIR spectral regions on PC1 for GY, with similar inverted behavior for GPC. Independently of the crop stages, the AUC approach based on VI temporal profiles demonstrated for all profiles weak relationships with GY (R^2^ *≤* 0.32) and very weak with GPC (R^2^ *≤* 0.1), whereby the most suitable VIs and the best temporal window for GPC assessment were identified.

### 3.4 Grain Protein Content Estimation Maps

**Figure 10:**
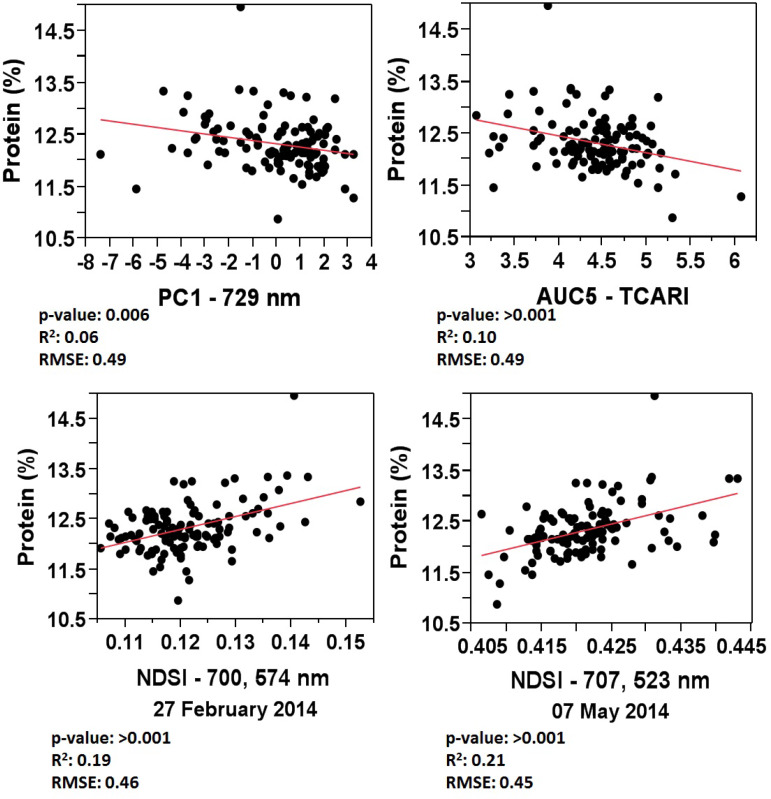
Regression plots between GPC and the best fittings of each exploratory approach, where *p*—value: *p*—values of the regression analysis; R^2^: coefficient of determination of the regression analysis; RMSE: root mean square error of the regression analysis.

To address the main focus of this study—the potential for timely GPC assessment for crop management as well as selective harvesting—and because grain protein comes at a higher aggregate value to farmers than yield, only the estimation of GPC is targeted in this section. From all the previously presented mono/multi—temporal spectral exploratory analysis methods, the result with the best fitting for GPC estimation was chosen from each method (Figure 10).

For the spectral exploratory analysis approach, the highest correlations with GPC were achieved using NDSI of a 700 and 574 nm combination from the 27 February image (R^2^: 0.19; RMSE: 0.46, root mean square error) and a 707 and 523 nm combination from the 7 May image (R^2^: 0.21; RMSE: 0.45). In the following figure, both are presented because the 27 February image provides information around the beginning of booting (early season), which is a suitable temporal window for crop management intervention targeting GPC correction. The 7 May image, which comes after physiological maturity (grain hard, late season), offers potential for selective harvesting strategies.

Within the multi—temporal exploratory analysis approach, the tested methods were tPCA and AUC of selected VIs. For the tPCA method, the 729 nm wavelength within PC1 obtained the highest correlation with GPC. The linear regression adjustment, although statistically significant at *p*—value < 0.05, showed an R^2^ of 0.06 and an RMSE of 0.49. For the AUC method, the TCARI index and MCARI/OSAVI ratio index of AUC5, which represents the area under the curve for the indices response from steam elongation (GS31) to anthesis (GS65), revealed the highest relationships with GPC (R^2^: 0.10; Table 6). Due to its number of used wavelengths and simplicity, just AUC5—TCARI (R^2^: 0.10; RMSE: 0.49) was selected for further presentation.

The comparison of relationships between GPC and the best fit of each exploratory analysis methods revealed (a) a general low to very low coefficient of determination (R^2^), (b) a general relatively high overall prediction error (RMSE) considering the low range variability of GPC for this field, and (c) that the spectral exploratory analysis approach surpasses the multi—temporal approach.

As outlined in the introduction, most remote and proximal sensing studies for GPC estimation were conducted on (a) fields and/or regions with naturally higher GPC variation [3,45,46] and/or (b) fields with different level treatments inducing higher GPC variation [2,3,27,47–50]. Generally, a higher range of calibration parameters allows better model adjustment for more precise prediction results. As an example, when studying relatively high GPC prediction accuracy, Wang et al. [46] demonstrated at the regional scale the potential of multi—temporal spectral image analysis using fused multispectral, broad—band satellite data for the crop growth period from jointing to anthesis (R^2^: 0.64). Although the overall prediction error of our study (RMSE: 0.45–0.49%) was relatively high for the conditions in the study area, the selected methods seem to be relatively more precise when compared to other proximal and remote sensing studies ([47], RMSE: 0.4–0.79%; [48], RMSE: 0.89–0.96%; [46], RMSE: 1.28–2.86%).

**Figure 11:**
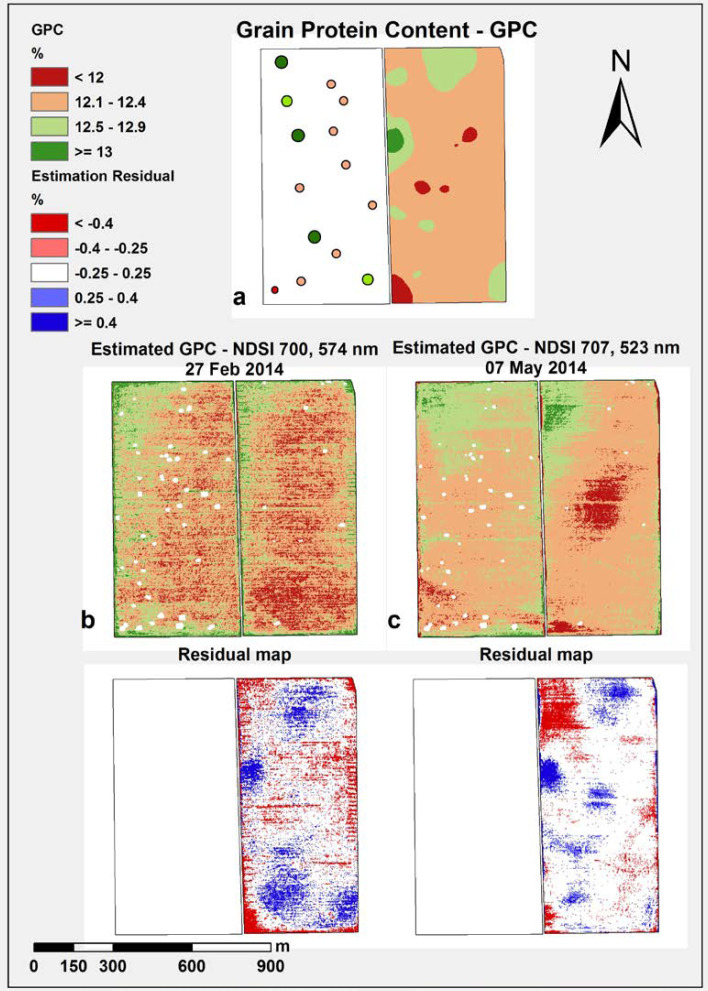
Measured interpolated GPC map (as shown in Figure 1)(**a**); estimated early—season GPC map and derived residual map (**b**); estimated late—season GPC map and derived residual map (**c**). White spots in the estimated maps are weed spots treated by the farmer.

Figure 11 depicts three GPC maps based on: (a) measured GPC data resulting from in—situ samples and laboratory analysis, (b) estimated GPC data resulting from NDSI 700/574 nm spectral combination of the 27 February image, (c) estimated GPC data resulting from NDSI 707/523 nm spectral combination of 7 May, and their respective residual maps (difference between the estimated map and the interpolated map using ground measurements).

For the detection of lower (<12.5%) and higher (*≥*12.5%) wheat grain quality areas within the field, both estimated maps show a very similar size and distribution of class zones, whereby higher GPC areas were estimated based on the late—season image. The late—season GPC estimation map reflects a more realistic situation of GPC at harvest time (as illustrated by the kriged GPC map based on in—situ measured data).

In general, the prediction quality parameters (R^2^, RMSE) indicated an overall low prediction potential, but without any specific information regarding the within—field variability. In contrast, the residual maps of Block B (Figure 11) give a spatially detailed picture of the prediction potential, making it possible to visualize the within—field variation of GPC. Using the NDSI 700/574 nm spectral combination of the 27 February image, 61.7% of the total field area could be mapped with relatively good prediction (residual class: −0.25–+0.25%) and an even larger field area (74.9% of total field area) using the NDSI 707/523 nm spectral combination from the 7 May image. Overestimations (residual class <−0.25%: 19.8% for February 27, 13.6% for 7 May) and underestimations (residual class >+0.25%: 18.5% for February 27, 11.4% for 7 May) were very similar for both NDSI combinations and their corresponding crop stage.

## 4 Conclusions

This research presents the evaluation of spectral and multi—temporal spectral exploratory analysis methods for the assessment of within—field variations of wheat GY and GPC using high—resolution hyperspectral airborne imagery.

For both parameters, all three tested exploratory analysis methods—(a) narrow—band physiological spectral indices (spectral approach), (b) temporal principal component analysis (multi—temporal approach), and (c) the integration of VIs over time (multi—temporal approach)—made it possible to identify the most valuable reflectance wavelengths matching wheat physiology as well as to detect the optimal time window across the crop cycle for image acquisition based on the most relevant crop stages for grain protein prediction. Moreover, this study revealed a generally low to very low coefficient of determination, whereby the spectral exploratory analysis approach surpasses the multi—temporal approaches. Considering the low range of GPC at the study site, the overall prediction error for GPC estimation is relatively high. However, compared to other proximal and remote sensing—based GPC assessment studies, the RMSE values from this study seem to be relatively low. Moreover, residual maps derived during the regression analysis of NDSIs and GPC proved to be good for within—field prediction assessment and that, in our study, an area of ~75% of the total field could be mapped with relatively good prediction.

For future studies, this study delivers valuable information on the use of remote sensing data for the assessment of within—field GY and GPC variability aiming N management and selective harvesting: (a) detecting the most important crop stage for image acquisition and (b) identifying the best reflectance wavelengths along the crop cycle. New studies targeting N management for grain protein should be conducted, specifically using long—term experiments with different varieties and locations and applying different amounts of N at different times to generate calibrations with RS data. Furthermore, investigating GPC variation over different scales using different proximal and remote sensing platforms and corresponding spectro—radiometers (e.g., hand—held and on—combine sensors, UAV, aircraft, satellite—based sensors) may enable transferring GPC prediction models from the field—scale to the regional scale.
